# The National Medical Union and the Insurance Acts

**Published:** 1916-08-19

**Authors:** 


### THE NATIONAL MEDICAL UNION AND THE INSURANCE ACTS.

### Memorandum.

EXPLANATORY NOTE.

The following Memorandum was furnished by the National Medical Union in response to an invitation received from the Secretary of the Commission of Investigation. The following members of the Union also attended before the Commission and gave evidence on the matters dealt with in the Memorandum:—Mr. V. T. Greenyer, F.R.C.S. (Chairman of Council), Dr. Alexander Morison, F.R.C.P. (Chairman of Executive Committee), and Drs. H. B. Greene, Edwin Smith, and G. D. Wilson (Members of Council). The taking of their evidence occupied some three and a-half hours, and the witnesses were questioned on many points. Among the matters dealt with were the evils of lay control, the refusal of choice of doctor, the inadequate medical service under the panels, the harmful influence of the present system of medical ethics and education, and various points such as the irregular distribution of the medical benefit surplus funds, and the sanction of "treatment" at the public expense by "herbalists" and other quacks. The National Medical Union is confident that good must come from an inquiry into the working of the Insurance Acts such as that now being carried on by the Commission of Investigation.

---

*Memorandum of Evidence on the working of the National Health Insurance Acts submitted by the National Medical Union to the Commission of Investigation now sitting at the House of Commons.*

The Medical Union advocates:—

(I.) **The Repeal of the Medical Benefit Section of the Insurance Acts,** on the ground that the panel system is an admitted failure, involving the following defects amongst others:—

(*a*) Pressure by committees, leading to the prescribing of cheap and inferior drugs and stock mixtures, with, in addition, what amounts to a pecuniary bribe (the "floating sixpence") to keep down the drug bill.

(*b*) Perfunctory attention, due to congested waiting-rooms and lack of time for examination of patients.

(*c*) Division of the medical profession, due to establishing a State monopoly granted to one section at the expense of the other.

(*d*) Extension of State grant for medical benefit to unregistered persons—*e.g.*, "herbalists," "bonesetters," and other quacks.

(*e*) Dissatisfaction of those insured persons who, preferring to employ the doctor of their choice, have in consequence to forfeit their medical benefit and pay twice. The panel doctors still receive capitation fees in respect of these persons, and have consequently a pecuniary interest in transferring their liability to non-panel doctors. They have a similar inducement to refer patients on their panel list to public and Poor-Law institutions.

(II.) **Lay Control is Objectionable to the Medical Profession,** and will always present a serious obstacle to an efficient service; for our calling has always been a free and liberal profession. Thus the attempt to control medical certification in the working of the Insurance Acts has led in Great Britain to constant friction, and in Ireland to a deadlock. Compulsory violation of professional secrecy is another evil for which lay control is responsible. The history of medical service under lay control has always been thoroughly bad.

(III.) **Free Choice of Doctor is Indispensable.**

(*a*) Mr. Lloyd George himself frequently emphasised the importance of granting free choice, until the Insurance Commissioners by regulation limited the choice to doctors on the panel.

(*b*) Permission to make "own arrangements" was arbitrarily refused by most Insurance Committees, notably those of the counties of London and Middlesex. This produced an unexpected "surplus medical benefit fund," being the proceeds of compulsory and inequitable contributions levied on insured persons declining to select a panel doctor. These contributions were distributed amongst the panel doctors by Insurance Committees against the repeated opinion of Mr. Danckwerts, K.C., expressly taken by the Insurance Committee of the County of London, that such distribution was "unlawful" and "in the nature of a dodge."

(IV.) **Adequate Treatment has not been Secured.**

(*a*) See No. I. (above).

For the latter portion of this article see p. 480.

### The National Medical Union and the Insurance Acts.

(*Continued from p.* 476.)

(*b*) Owing to insufficient sanatorium and tuberculosis dispensary accommodation, numbers of patients are sent to Union workhouses. In the County of London there are only 300 beds for tuberculous insured persons. Domiciliary treatment for tuberculosis is also most unsatisfactory, and often criminally culpable.

(*c*) There is no provision under the Acts for consultations, anæsthetics, operations (even those of a minor character), or for special treatment or diagnosis—*e.g.*, eye, ear, and throat cases, *x*-ray examination, etc.

(*d*) Necessary medication and appliances are withheld for fear of a surcharge for their prescription.

(V.) **Professional Ethics have been Ignored.**

(*a*) Medical men who in some instances are panel doctors have been appointed referees by Approved Societies and paid by them. These referees visit patients, diagnose their ailments, and often discredit the certificates and opinions of the regular medical attendants, subsequent events proving in some cases that an obvious injustice to the patient has been done.

(*b*) There has been, under State compulsion, a wholesale transference of the patients of private practitioners to the lists of panel doctors.

(*c*) Contract practice in any form, leading as it does to collective bargaining and undercutting, has always been and will remain unacceptable to a very large section of the profession.

(VI.) **Insurance has not been Restricted to those Equitably Entitled to it.** Highly-paid manual labourers, such as engineers' mechanics and skilled artisans, have neither the need nor often the desire for the compulsory insurance to which they are now subjected. The same objection applies to the inclusion of the dependants of well-to-do persons.

In conclusion, the National Medical Union has never been opposed to the application of the principle of National Health Insurance to the necessitous classes of the community. The above Memorandum is furnished in response to the invitation of the Commission of Investigation of the Faculty of Insurance, and indicates some of the grounds upon which exception is taken to the existing scheme and administration of the medical benefit section of the National Health Insurance Acts.

